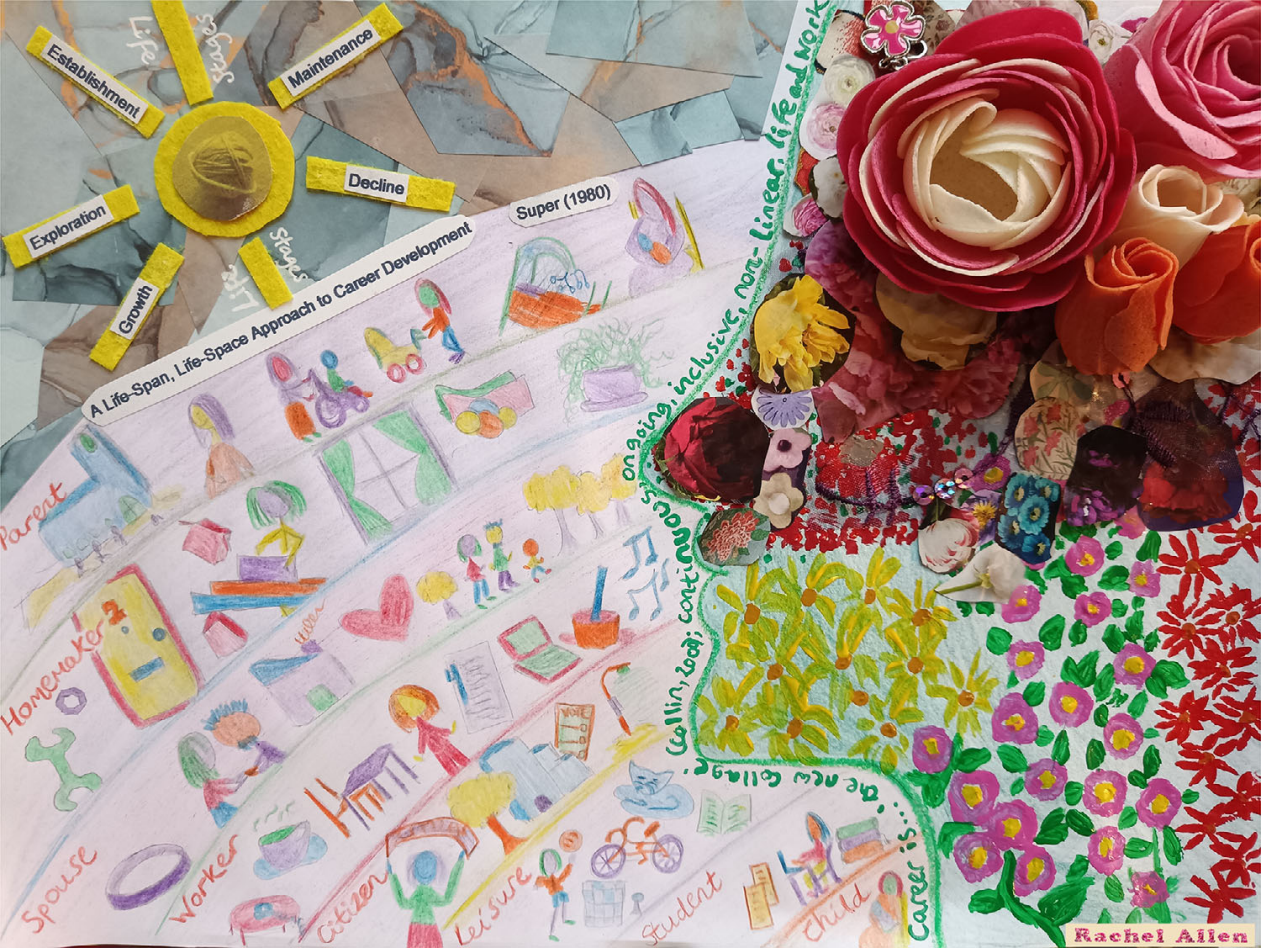# Young‐Onset Dementia and Career Development ♥ An Artsract

**DOI:** 10.1002/alz.084014

**Published:** 2025-01-09

**Authors:** Rachel Louise Allen

**Affiliations:** ^1^ University of the West of Scotland, Paisley, Scotland United Kingdom

## Abstract

My PhD research is about Young‐Onset Dementia and Career Development, focusing on women’s experiences.

This artwork or abstract (‘artstract’) represents how the research has developed to date. Understanding of career is informed by Super’s (1980) Life‐Span, Life‐Space approach to career development, which can be understood flexibly to incorporate diverse experiences (including those of women and those living with a dementia diagnosis during working age).

The sunshine, represented here as a ball of yarn (because yarn can be useful but can also unravel), represents the life stages of the approach, with the rainbow stripes below reflecting the Life Rainbow (Super, 1980). The woman’s face in silhouette (so the whole self cannot be seen) is decorated with painted flowers, building to a 3D representation around the brain and senses. This reflects the new beginnings and opportunities that can arise when living with dementia. However, several layers of paint, fabric and magazine collage are not visible because dementia can erode memories and abilities that the person can no longer utilise.

The ’mess’ of collage and materials mixed with the simpler pencil lines represents the complexity of career. Whereas once this could be considered perhaps as linear, relating only to paid work and progression, this research conceptualises career more as life and work. When a woman is diagnosed with young‐onset dementia, this could impact several life stages and multiple roles in her life. She may be employed or retired, a carer for children, grandchildren or aging parents, and active in her community. Dementia is a condition with stigma, and this artstract challenges stereotypical assumptions and representations of dementia as something that only affects older people, or that those living with dementia do not have a career or that career cannot continue whilst living with a diagnosis. The bright colours were chosen to reflect the Life Rainbow (Super, 1980) and emphasise the experience of hope whilst living with a diagnosis.

Super, Donald (1980) **A Life‐Span, Life‐Space Approach to Career Development,** Journal of Vocational Development 1980 Vol. 16 Pages 282‐298